# News Media Framing of Suicide Circumstances and Gender: Mixed Methods Analysis

**DOI:** 10.2196/49879

**Published:** 2024-07-03

**Authors:** Jasmine C Foriest, Shravika Mittal, Eugenia Kim, Andrea Carmichael, Natalie Lennon, Steven A Sumner, Munmun De Choudhury

**Affiliations:** 1 School of Interactive Computing Georgia Institute of Technology Atlanta, GA United States; 2 National Center for Injury Prevention and Control Centers for Disease Control and Prevention Atlanta, GA United States; 3 Accenture Arlington, VA United States

**Keywords:** suicide, framing, disparities, reporting guidelines, gender, stigma, glorification, glorify, glorifying, suicidal, self harm, suicides, stigmatizing, stigmatization, disparities, reporting, news, journalist, journalists, journalism, machine learning, NLP, natural language processing, LLM, LLMs, language model, language models, linguistic, linguistics, reporter, reporters, gender, digital mental health, mHealth, media

## Abstract

**Background:**

Suicide is a leading cause of death worldwide. Journalistic reporting guidelines were created to curb the impact of unsafe reporting; however, how suicide is framed in news reports may differ by important characteristics such as the circumstances and the decedent’s gender.

**Objective:**

This study aimed to examine the degree to which news media reports of suicides are framed using stigmatized or glorified language and differences in such framing by gender and circumstance of suicide.

**Methods:**

We analyzed 200 news articles regarding suicides and applied the validated Stigma of Suicide Scale to identify stigmatized and glorified language. We assessed linguistic similarity with 2 widely used metrics, cosine similarity and mutual information scores, using a machine learning–based large language model.

**Results:**

News reports of male suicides were framed more similarly to stigmatizing (P<.001) and glorifying (P=.005) language than reports of female suicides. Considering the circumstances of suicide, mutual information scores indicated that differences in the use of stigmatizing or glorifying language by gender were most pronounced for articles attributing legal (0.155), relationship (0.268), or mental health problems (0.251) as the cause.

**Conclusions:**

Linguistic differences, by gender, in stigmatizing or glorifying language when reporting suicide may exacerbate suicide disparities.

## Introduction

### Background

Suicide is a leading cause of death in the United States, claiming over 48,000 lives in 2021 [1]. Rates of suicide and self-harm are significantly different for males and females. In 2021, US age-adjusted suicide rates were higher among males (22.8/100,000) than females (5.7/100,000) [1]. However, reported rates of nonfatal self-harm in 2020, the most recent year for which such data are available, were higher among females (189.4/100,000) than males (123.8/100,000) [2].

### Gender and Suicide

The observed gender disparities in suicide death rates between males and females are echoed in gender differences in other suicide-related behaviors such as ideation, attempts, and mechanisms [3]. Women have higher rates of lifetime suicidal ideation [4] and suicide attempts [5]. Wang et al [3] also report that men experience higher rates of suicide likely due to the increased lethality of the mechanism. Circumstances of suicide, or negative life events that precipitate suicide-related behavior [6], also vary among men and women according to the analysis of suicides in the National Violent Death Reporting System [6]. For example, previous analysis correlates negative financial and employment circumstances with increased male suicides in 2009 [7]. Suicide-related behavior is therefore highly gendered, and research that seeks to inform understanding and prevention of suicide must consider gender as a layer of analysis. While we acknowledge the call for expanded US research on gender differences across all suicide-related behaviors [8], this study includes gender as an essential layer of analysis to understand reports of suicide deaths.

### Unsafe Media Reporting about Suicide

Unsafe news reporting is among many risk factors that contribute to suicide [9]. For example, studies have shown that sensationalized reporting, including that of celebrity suicides, is positively correlated with a rise in suicides [10,11]. Other research has shown that specific characteristics of news coverage, for example, descriptions of an epidemic or suicide myths, may also shape population-level effects on suicide [12]. However, the need to better understand how specific language in framing suicide news shapes its perception is ongoing. In particular, one critical research need is to better elucidate the differences in unsafe reporting by gender when considering the differences in suicide-related behavior. While discussions of all suicide-related behavior are likely subject to unsafe reporting, this study aligns with the focus of existing work examining unsafe media portrayals of individual suicide deaths in particular.

### Stigma and Glorification in News Reports

The suicide reporting guidelines detail best practices for news organizations to safely report suicides and generally focus on content that should not be included, such as the mechanism or specific location of suicide [13]. However, assessing subtle nuances about how suicide may be discussed or framed in other harmful ways is more challenging. In this study, we explore two particular ways of framing suicide in news reports, which are stigmatizing and glorifying descriptions.

Although elucidating the precise language that stigmatizes or glorifies suicide is challenging, researchers have developed the validated Stigma of Suicide Scale (SOSS) [14], which helps to linguistically define these constructs as detailed below. The SOSS furthers research in this area by measuring specific words and how they may stigmatize or glorify perspectives. The scale reports a list of words that can be used to further understand, identify, and explore such framings. For example, “shallow,” “pathetic,” and “immoral” are associated with stigmatizing perspectives (ie, those perspectives that ascribe a negative attitude or perception toward those who die by suicide). Similarly, “understandable,” “brave,” and “motivated” are associated with glorifying perspectives (ie, those perspectives that seek to normalize suicide) [14].

### Identifying Problematic Framing

Carefully framing suicide deaths in the news media is essential as the language people use shapes perceived reality [15] and can influence suicide-related behavior [12]. Previous articles on suicide reporting have mostly focused on assessing the degree of explicit adherence to elements of the suicide-safe reporting guidelines [16]. While recent articles have applied machine learning and natural language processing to suicide news, they have largely focused on using these techniques to automate identifying more structured elements present in the suicide reporting guidelines [13]. Thus, in this research, we aim to develop natural language processing methods to measure and explore the more nuanced problematic framings of suicide using stigmatizing or glorifying language. Furthermore, we explore such framings by gender and by the circumstance of suicide. We pose two main research questions: (1) how does the overall framing of suicide differ by gender identity? and (2) how does such framing differ by gender when considering the specific circumstance of suicide?

## Methods

### Data Collection

Data for this study were comprised of news articles from All the News [17], a leading benchmark data set used in the field of computer science and natural language processing–based news research; the public availability of the data set allows for independent validation of results by the academic community and facilitates transparent head-to-head comparison of different natural language processing approaches. The data set includes 143,000 publicly accessible articles published by 15 print and digital American publications, from 2015 to 2017. Articles included in this data set are in English and come from publishers across political alignments. Examples of publishers include the New York Times, CNN, Washington Post, Breitbart, Fox News, Buzzfeed News, and others [17]. We identified articles from this data set relevant to suicide using suicide-related keywords previously curated by public health experts [18]. Previous research has used the All the News data set for identifying political polarization [19], differences between human- and machine-generated news [20], and news recommendations [21]. We randomly sampled 800 suicide-related articles, in English, and excluded 600 articles mentioning homicide-suicide, suicide bombings, suicide attempts, suicide euphemisms, and fictional portrayals of suicide [22], such as those illustrated in fictional books, television, and movies, as these classifications in media are researched differently in suicidology. In total, 200 articles met the inclusion criteria of mentioning a real-life suicide death not involving the homicide of another person and constitute the data set in this study.

### Qualitative Methods

We used 2 coders for a deductive approach to annotate article mentions of gender and circumstances of the suicide to answer our research questions. We derived our definitions of circumstances from Chancellor et al’s [23] validated annotation scheme of risk factors for suicide. Chancellor et al [23] developed the scheme using social media data of 200 Reddit community posts from r/SuicideWatch with a focus on construct validity. This scheme offers a formal technique to identify clinical suicide–related information in lay contexts.

### Quantitative Methods

#### Framing—Stigma of Suicide Scale

As noted above, we derived the frames of stigmatization and glorification from the SOSS study [14]. This validated scale consists of linguistic descriptors of suicide. To derive the scale, Batterham et al [14] used principal component analysis of survey results from the public, rating 80 one-word descriptors of someone who dies by suicide to produce a list of words associated with stigmatization and glorification of suicide. The SOSS includes a third frame of isolation or depression (ie, “unhappy,” “depressed,” and “sad”), which we briefly report in this study but do not focus on the subsequent analyses as we found no discernable differences between genders, perhaps because depressed mood is a common symptom preceding suicide [24], and such language can be applied generically.

#### Linguistic Representation of the Stigma of Suicide Scale

To get a comprehensive linguistic representation of stigmatizing and glorifying language as represented by the SOSS, we used natural language modeling techniques instead of relying on lexicon-based approaches that restrict themselves to finding exact matches of a fixed set of keywords. We adopted language modeling techniques to better capture linguistic context and nuanced writing style, considering semantic and syntactic relationships between words and phrases in sentences [25,26]. Existing research has shown that such context-based approaches outperform lexicon-based ones on natural language understanding tasks [27,28]. We used the keywords identified by Batterham et al [14] for stigmatization and glorification to generate vector representations for each frame. Vector representations are large numerical representations of text used in natural language processing, and the numerical representations help identify other words that tend to co-occur or lie in the vicinity of particular words of interest. We used Bidirectional Encoder Representations from Transformers (BERT; Google) [29] along with their relevant extracted synonyms using WordNet (Princeton University) [30], a large English database containing synonymous groups (synsets) of nouns, verbs, adjectives, and adverbs. BERT is a leading large language model that is pretrained on billions of documents from the internet and encodes text to generate word-embedding representations for all the words or phrases associated with a SOSS dimension. Adding representations of synonyms strengthens the semantic representation of SOSS dimensions. After this addition, we manually examined the dictionaries to remove irrelevant words captured by WordNet. We calculated Cohen κ to assess interrater reliability (IRR) for each SOSS dimension. Finally, we averaged the relevant individual word embeddings to generate the final vector representation of each SOSS dimension.

#### Representation of News Articles

We generated a unique vector representation for each news article in our data set to answer research question 1 and to analyze how suicide is framed using the SOSS dimensions. We again used BERT, programmed in Python (Python Software Foundation) using Hugging Face transformers [31], to extract a sentence-level embedding representation for each news article. This allowed us to capture nuanced attributes such as context, framing and writing style, and the relationship between words in a sentence. Note that we did not aggregate the individual embeddings of all the words present in a news article to generate its linguistic representation. Sentence-level BERT embeddings ensured that we captured relevant dependency between words used in the news article. As a result, each news article was characterized using a vector representation to compare it against the SOSS dimension embeddings.

#### Suicide Framing Based on the Axis of Gender

We analyzed how suicide is framed in the news based on the victim’s identified gender by comparing the embedding representation of each news article with the 3 vectors of the 3 SOSS dimensions (stigmatization, glorification or normalization, and isolation or depression). We adopted the cosine similarity scoring framework, as used in previous work [32], to score each article in our data set against the SOSS dimensions. These cosine similarity scores range from 1 to 1, where a higher positive score represents a stronger alignment between a news article and the corresponding SOSS dimension.

Further, we divided all the articles into groups based on the annotated gender identity of the victim. Toward inclusive representation of gender identity in our analysis, we annotated for cisgender, where one’s gender identity aligns with their assigned sex, and transgender, where one’s gender identity does not align with their assigned sex [33]. This resulted in the ultimate formation of 2 groups corresponding to the most represented gender identities in our data set, which are female and male. Note that some news articles made references to multiple victims. To handle such cases, articles mentioning same-gender identity victims were treated as reporting a single female or male death, and those with mixed-gender mentions were processed by manually extracting relevant text for each victim, respectively, to inform the analysis.

### Ethical Considerations

The study did not meet the criteria for institutional research board review as it did not involve interactions with human or other living subjects, private or personally identifiable data, or any pharmaceuticals or medical devices [34]. The data set is comprised only of public, open-source news articles. While this data set is public, widely used, and institutional research board exempt, all possible care was taken to ensure the results of this study were communicated sensitively and safely given the context of suicide.

## Results

### Overview

A total of 221 real-life suicide deaths were reported from the 200 articles. We identified almost 70% (68.4%, 153/221) of these deaths to be cisgender male, 28.4% (64/221) cisgender female, and 0.4% (1/221) were identified as transgender male. Less than 2% (3/221) of deaths were reported without specifying the decedent’s gender. The main analysis focused on articles reporting suicide deaths of cisgender men and women due to the underrepresentation of noncisgender identities in the sample. This paper targets the circumstances of suicide, more than one of which can be attributed to a single death and characterizes them in the results that follow.

### Circumstances of Suicide

In total, 153 male decedents in our study had 159 circumstances and 64 female decedents had 75 circumstances (Table 1). We found 21 unique circumstance types among our sample of articles. Analyses described in this paper characterize the top 7 circumstances (Table 1) that occurred most frequently, which are (1) unspecified (n=60), (2) legal problem (n=41), (3) explicit statement of mental health symptoms or diagnosis (n=38), (4) social or relationship problem (n=35), (5) physical health problem (n=33), (6) financial or job problem (n=19), and (7) preceding suicidality (n=13).

**Table 1:** Summary of top 7 circumstances of suicide by gender annotated in 200 news articles.

Gender	Unspecified, n (%)	Legal problem, n (%)	Mental health symptoms or diagnosis, n (%)	Social or Relationship, n (%)	Physical health, n (%)	Financial or job, n (%)	Preceding suicidality, n (%)	Full sample, n
Cisgender men	35 (22)	31 (19.5)	29 (18.2)	21 (13.2)	24 (15.1)	10 (6.3)	9 (5.7)	159
Cisgender women	24 (32)	9 (12)	8 (10.7)	13 (17.3)	8 (10.7)	9 (12)	4 (5.3)	75
Transgender^a^	0 (0)	0 (0)	0 (0)	1 (100)	0 (0)	0 (0)	0 (0)	1
Unspecified^a^	1 (25)	1 (25)	1 (25)	0 (0)	1 (25)	0 (0)	0 (0)	4

^a^Excluded from additional analyses due to underrepresentation in the data set.

### Framing Suicide by Gender

**Figure 1:**
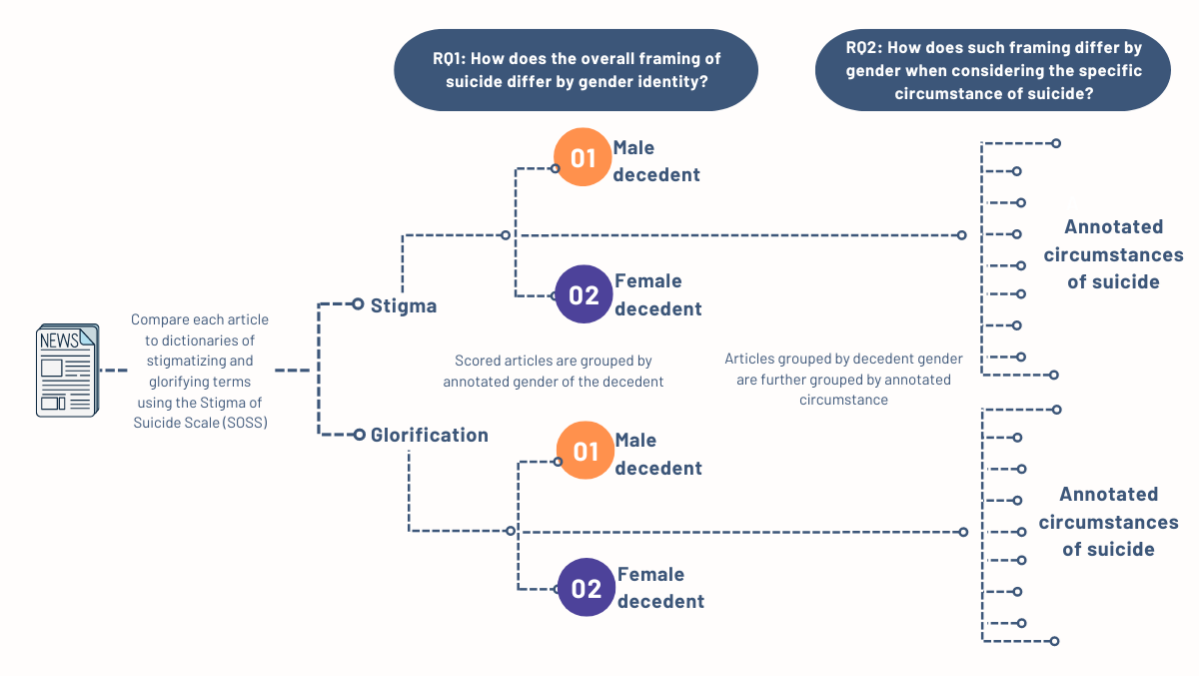
Illustration of the relationship between research questions and analysis.

**Figure 2:**
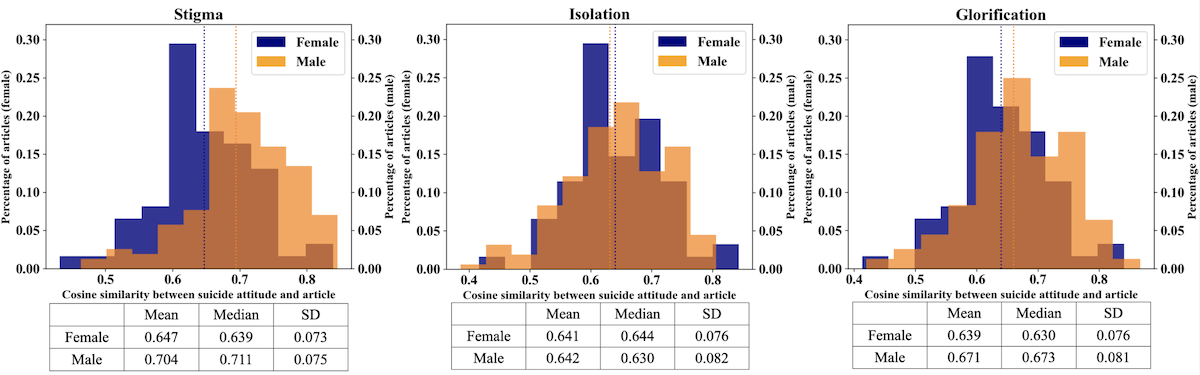
Percentage distribution plots of cosine similarity scores by gender compared to the Stigma of Suicide Scale dimensions.

The distributions in Figure 1 illustrate the proportion of articles reporting a male (orange) or female (blue) suicide death with linguistic overlap with each SOSS attitude of stigma, isolation or depression, and glorification. In Figure 2, and subsequent figures, dotted lines represent mean cosine similarity scores across the 2 genders that are male (orange) or female (blue). The isolation or depression frame is excluded from the remaining analyses as no difference occurred in cosine similarity. Cosine similarity measures how similar 2 vectors are with increasing values representing increased similarity. A Mann-Whitney *U* test revealed that articles reporting male suicide deaths contained statistically significant more linguistic overlap with the stigma attitude on average than papers reporting female suicide deaths (*P*<.001). Such articles reporting male deaths also contained statistically significant more linguistic overlap with the glorification attitude on average than articles reporting female suicide deaths (*P*=.005). We found no statistically significant difference in the average linguistic similarity between papers reporting male or female suicide deaths (*P*=.07) regarding the isolation attitude.

Assessing IRR using Cohen κ for each SOSS dimension demonstrated acceptable reproducibility, ≥75%, between coders with scores of 0.84, 0.89, and 0.94 for stigma, isolation or depression, and glorification, respectively. Assessing IRR using Cohen κ for decedent gender and circumstance demonstrated acceptable reproducibility (≥75%) between coders with a score of 0.81.

### Framing Circumstances of Suicide by Gender

We reported mutual information (MI) scores, which are widely used in the information theory literature, to determine differences by gender in stigmatizing or glorifying language as used under a particular circumstance of death. MI is an indicator of similarity (score farther from 0) or dissimilarity (score closer to 0) between two distributions [35]. In Table 2, the MI scores compare articles reporting male and female suicides to the stigma and glorification of SOSS attitudes for the 7 most frequently reported circumstances in the sample. Regarding stigmatizing language, reports of male and female suicide deaths were least similar when attributed to a legal problem (0.155), a social or relationship problem (0.268), or when no circumstance is described (0.312). Regarding glorification language, reports of male and female suicides were least similar when attributed to a legal problem (0.132), an explicit statement of mental health symptoms or diagnosis (0.251), or a physical health problem (0.320).

**Table 2:** Mutual information scores between male and female suicide deaths by circumstance. All the News data set—United States, 2015–2017. The closer the score is to 0, the less mutual information is present between the groups.

Circumstance	Stigma MI^a^	Glorification MI
Unspecified	0.312^b^	0.578
Legal problem	0.155^b^	0.132^b^
Explicit statement of mental health symptoms or diagnosis	0.421	0.251^b^
Social or relationship problem	0.268^b^	0.522
Physical health problem	0.443	0.320^b^
Financial or job problem	0.349	0.849
Preceding suicidality	0.343	0.441

^a^MI: mutual information.

^b^Circumstance with the least linguistic similarity between males and females for the stigma and glorification frame is further characterized in the text.

**Figure 3:**
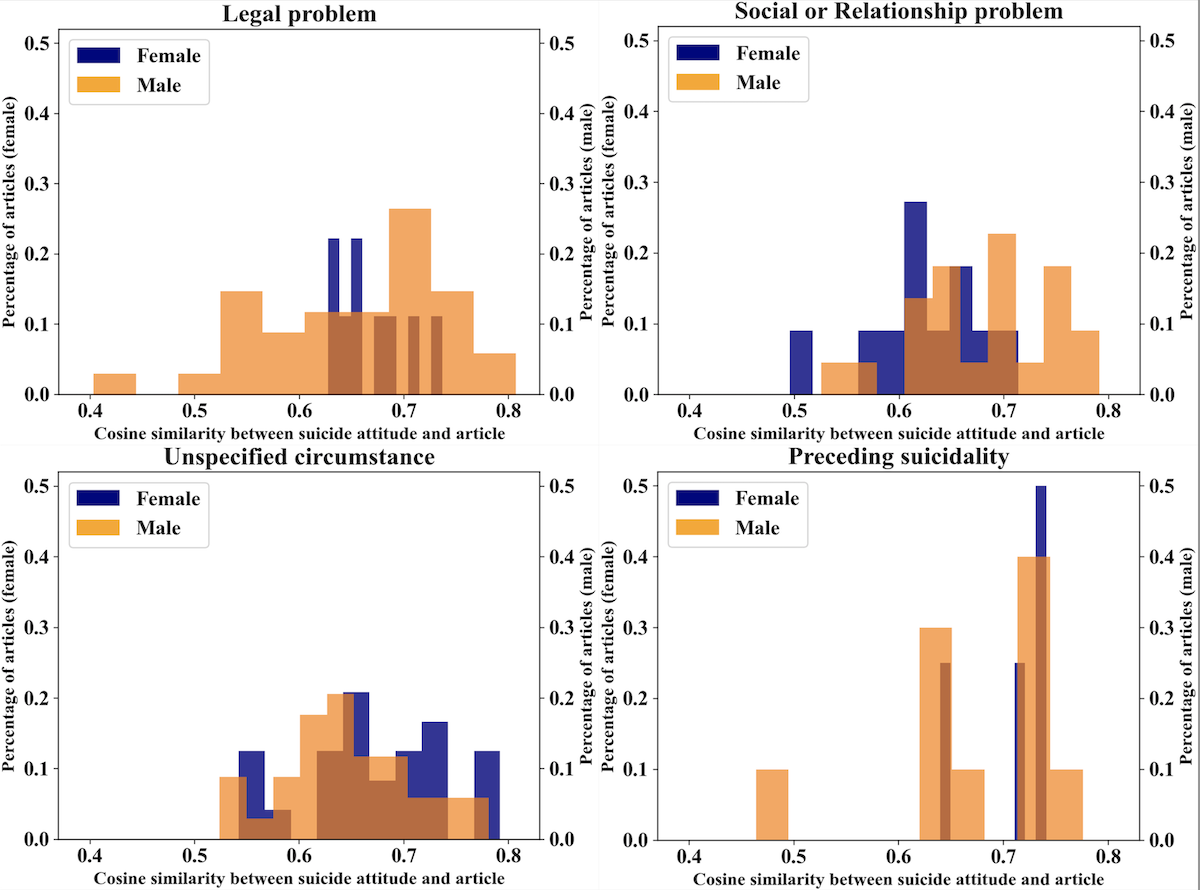
Percentage distribution of articles by gender and circumstance compared to the attitude of “stigma”.

Article similarity to the stigma attitude for the circumstances with the greatest difference in MI is displayed in Figure 3. Mean cosine similarity measurement revealed that reports of female suicides attributed to a legal problem (0.671) or without any circumstance specified (0.673) were more linguistically similar to stigmatizing framing than reports of male suicides attributed to the same circumstances (Figure 3). However, male deaths attributed to a social or relationship issue (0.682) had greater linguistic similarity to stigmatizing framing than reports of female deaths of the same circumstance (0.624).

**Figure 4:**
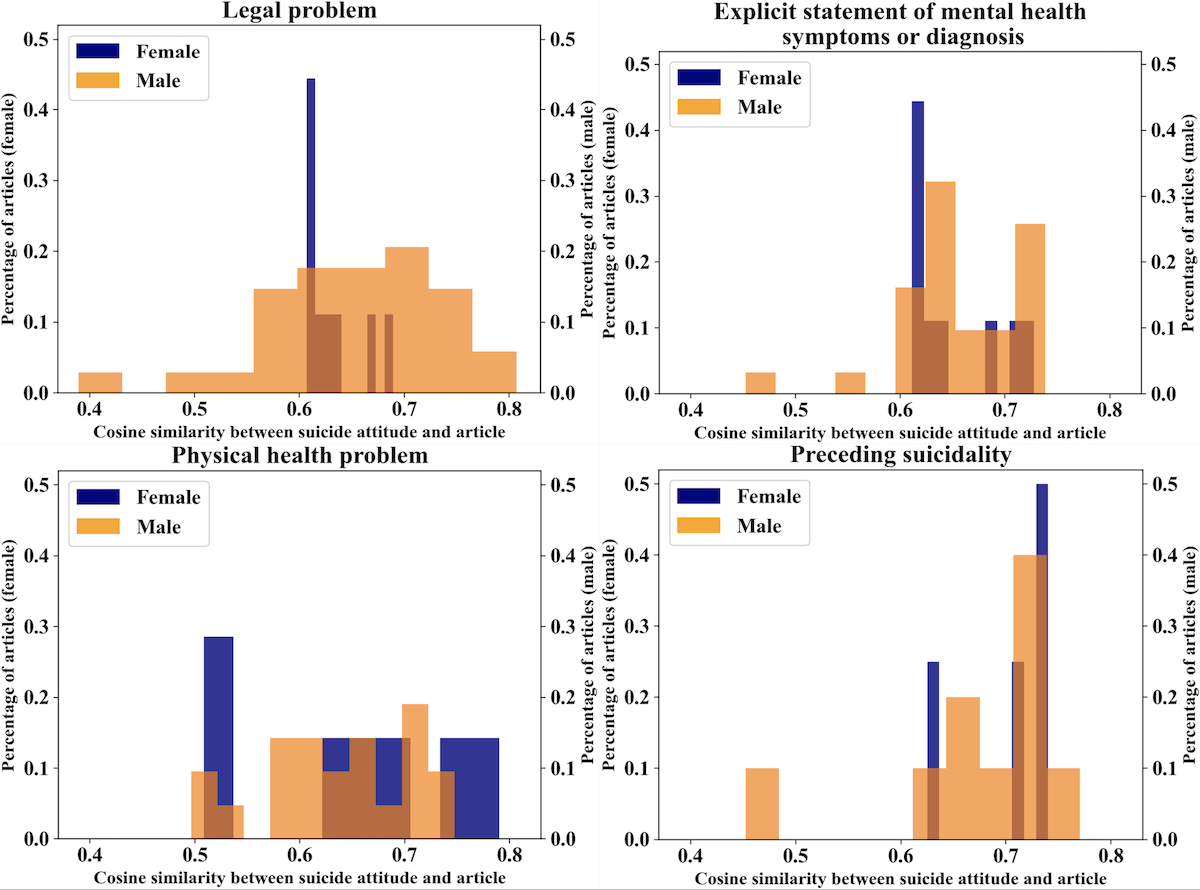
Percentage distribution of articles by gender and circumstance compared to the attitude of “glorification”.

Similar results hold for the circumstances with the least MI by gender regarding the glorification attitude. On average, reports of male suicides attributed to a legal problem (0.654), mental health symptom or diagnosis (0.657), or a physical health problem (0.639) were more linguistically similar to the glorification attitude than those reporting female suicides (Figure 4).

Suicidality had the fourth lowest MI score for both the stigma (0.343) and glorification (0.441) attitudes. The difference between the distributions of articles for this circumstance was less than the other circumstances; the linguistic similarity of reports of these suicides both to the stigma (Figure 3) and glorification (Figure 4) attitudes was higher than for other circumstances, regardless of decedent gender. The average cosine similarity to the glorification attitude for the suicidality circumstance was 0.702 for females and 0.656 for males in Figure 4. The average cosine similarity to the stigma attitude for suicidality as a circumstance was 0.708 for female deaths and 0.671 for male deaths.

## Discussion

### Key Findings

This study describes the reported gender of decedents and the circumstance of suicide in news articles and provides evidence that linguistic framing differs by both gender and circumstance.

Our comparison of SOSS attitudes in news articles revealed that descriptions of male suicides, on average, had higher linguistic scores for stigmatization and glorification than female suicides. This finding is important considering the observation that males die by suicide at higher rates than females in the United States, largely as a consequence of the greater use of firearms in suicide attempts [3]. Stigmatizing language, which can reduce help-seeking behavior [36], and language that glorifies or normalizes suicide acts [37], which may induce suicide-related contagion [38], are thereby concerning aspects of media reporting on suicide. This merits further attention as part of efforts to reduce high rates of suicide among males and other populations in need.

### Circumstances of Suicide and Stigmatizing Language

We used MI scores to identify differences in stigmatizing and glorifying language by gender and circumstance, discerning for which groups these differences were most pronounced. The scores indicated that the greatest differences, by gender, in stigmatizing language were those that discussed a legal problem, social or relationship problem, or no specified circumstance.

Language in reports of female deaths attributed to legal problems had higher linguistic similarity to attitudes of stigma than reports of male deaths of the same circumstance. However, the inverse was true for male deaths attributed to a social or relationship problem. The proportion of articles in our sample attributing legal and social or relationship problems as circumstances to each gender is opposite of the proportions of disclosed circumstances in the National Violent Death Reporting System [6]. Increased stigmatizing language in reports of women experiencing legal problems and men experiencing relationship problems may be attributed to their perceived deviation from social norms [39]. Reports of female deaths not attributed to any circumstance were more linguistically similar to the stigma attitude than reports of male deaths—demonstrating the presence of discernable stigmatizing language even without specifying the circumstances of suicide. This shows that reporting suicide in more general details, per the safe reporting guidelines, may not eliminate stigmatization when framing death.

### Circumstances of Suicide and Glorification Language

Similarly, when examining attitudes of glorification, news articles contain different language when reporting suicide deaths of men and women. We observed the greatest differences in language reflecting attitudes of glorification in articles that attributed death to a legal problem, explicit statement of mental health symptom or diagnosis, or a physical health problem. Specifically, reports of male deaths attributed to these problems had higher linguistic similarity to attitudes of glorification than those of female deaths for the same circumstances. Identifying these circumstances as being prone to using glorifying language for male suicide decedents may offer insights into areas where greater disparity exists in safe reporting by gender for more focused education among news organizations and their staff. For example, a correlation study of the SOSS used for this analysis indicates that male gender among respondents is correlated with decreased suicide literacy and increased stigma and glorifying attitudes of those who die by suicide [40]. As a 2021 study finds a greater stigma of suicide among medical professionals compared with the public [41], news publishers should consider education to increase suicide literacy among journalists.

Again, it should be noted that for a sizeable minority of deaths (22% for males and 32% for females), there was no specified circumstance. This may partly reflect the effect of the safe suicide reporting guidelines, which advise against oversimplifying or speculating the reason for the suicide [13]. Still, we find that harmful language persists in the absence of unsafe details.

### Shoring Up Safe Reporting of Suicide

This work brings light to the knowledge about the potential hazards of new reports of suicide beyond existing safe reporting guidelines. Current guidelines encourage the omission of specific details from journalistic reporting of suicide deaths such as method, location, or personal details of the decedent [13]. The relationship between adherence to safe reporting guidelines and suicide prevention is well established [16,42,43]. However, our findings suggest that stigmatizing and glorifying framing, facilitated by harmful language, persists in news reports of suicide regardless of adherence to safe reporting guidelines. This is especially exhibited by the presence of such language even when a circumstance is not attributed to the suicide death (Figure 3). Previous work highlights that stigmatizing and glorifying language has implications on public perceptions of suicide [44-47] and prevention strategies of help-seeking [36,37]. Yet, strategies to mitigate such language in the reporting of suicide deaths could be expanded upon in contemporary guidelines [13].

In response to this gap, we call on future work to investigate the relationship between guideline adherence and harmful framing in news reports of suicide deaths and all other suicide-related behaviors. Such investigation informs the extension of existing journalistic guidelines to include unsafe language. The use of computational approaches in this study illustrates the feasibility of this task. Such methods may support the development of tools to detect the harmful language in news reporting of suicide as demonstrated in a similar recommendation of 3 models of artificial intelligence tools to improve adherence to safe reporting guidelines [48] as a key prevention strategy.

### Additional Implications

Although gender minorities experience elevated risk and rates of suicide [9], our data do not allow us to discern if news media reports of suicide reflect these disparities or to compare reporting language between them. Gender minorities were underrepresented in this sample of suicide reports in the news even when the information was available. Existing research that explores suicide and gender focuses on risk factors for men and women [49]. This focus may be influenced by limited disclosure of nonconforming gender identity or default reporting of sex assigned at birth, often precluding public health surveillance [49]. Facing the same challenge, this work explored framing language in news reporting across all gender identities. Still, the absence of these identities in news reports prevents a complete understanding of stigmatizing language in these populations.

Our analysis revealed an unexpected finding. While we did not detect a discernable difference, by gender, in the linguistic similarity to stigma or glorification regarding the circumstance of preceding suicidality, reports of suicide attributing to this circumstance displayed greater similarity to both stigmatizing and glorifying attitudes compared with reports attributed to other circumstances. This suggests that a history of suicidal behavior is often reported in an unsafe way.

### Limitations

Limitations of this study include its sample size, our capacity to describe or account for the audience consuming news from the venues analyzed from this American outlet–focused data set, the age of the articles, and the lack of availability of some sociodemographic attributes in annotated articles, all of which may preclude generalizability of the findings. Although the study team intended to annotate for age, race, and occupation in addition to gender, recognizing that identity is intersectional, these details were largely unavailable to inform the analysis, possibly due to adherence to the safe reporting guidelines. Although gender was available in the sample, gender minorities were underrepresented. Considering larger and more diverse corpora of news may allow for deeper analysis based on an intersectional lens.

### Conclusions

Previous studies have investigated stigma and framing in news reports of suicide [47] or have focused on specific stigmatizing suicide terms [50]; however, this study examined suicide reporting language using a state-of-the-art natural language processing approach and revealed important differences by gender, and particularly stigmatizing and glorifying language for specific circumstances.

Safe reporting and messaging following a suicide is an important approach that can be used to lessen harm and reduce the future risk of suicide [51]. Journalistic adherence to media guidelines can prevent imitative suicide [42]; however, further opportunities exist to understand which elements of language, such as the 2 on which we focused in this study, are the most impactful [52]. While information management strategies such as those prescribed by the safe reporting guidelines seek to prevent harmful suicide reporting [53], actual efficacy and fidelity of adherence may differ based on the sociodemographic characteristics of the decedent, such as gender. The subtle, yet harmful framing of who experiences suicide in news media might influence prevention, intervention, and help-seeking behaviors [36], particularly among already disparaged or marginalized populations. Identifying sociodemographic differences in such framing supports the need to further understand suicide disparities and develop tailored stigma reduction strategies.

## Abbreviations

BERT: Bidirectional Encoder Representations from Transformers
CDC: Centers for Disease Control and Prevention
IRR: interrater reliability
MI: mutual information
SOSS: Stigma of Suicide Scale

## References

[1] (2021). About multiple cause of death, 1999-2020. Centers for Disease Control and Prevention.

[2] (2011). WISQARS (Web-Based Injury Statistics Query and Reporting System).

[3] Wang J, Sumner SA, Simon TR, Crosby AE, Annor FB, Gaylor E, Xu L, Holland KM (2020). Trends in the incidence and lethality of suicidal acts in the United States, 2006 to 2015. JAMA Psychiatry.

[4] Schrijvers DL, Bollen J, Sabbe BG (2012). The gender paradox in suicidal behavior and its impact on the suicidal process. J Affect Disord.

[5] Bommersbach TJ, Rosenheck RA, Petrakis IL, Rhee TG (2022). Why are women more likely to attempt suicide than men? Analysis of lifetime suicide attempts among US adults in a nationally representative sample. J Affect Disord.

[6] Chen T, Roberts K (2021). Negative life events and suicide in the National Violent Death Reporting System. Arch Suicide Res.

[7] Chang S, Stuckler D, Yip P, Gunnell D (2013). Impact of 2008 global economic crisis on suicide: time trend study in 54 countries. BMJ.

[8] Carretta RF, McKee SA, Rhee TG (2023). Gender differences in risks of suicide and suicidal behaviors in the USA: a narrative review. Curr Psychiatry Rep.

[9] (2022). Centers for Disease Control and Prevention. Disparities in Suicide.

[10] Phillips DP (1974). The influence of suggestion on suicide: substantive and theoretical implications of the Werther effect. Am Sociol Rev.

[11] Sinyor M, Tran US, Garcia D, Till B, Voracek M, Niederkrotenthaler T (2021). Suicide mortality in the United States following the suicides of Kate Spade and Anthony Bourdain. Aust N Z J Psychiatry.

[12] Niederkrotenthaler T, Voracek M, Herberth A, Till B, Strauss M, Etzersdorfer E, Eisenwort B, Sonneck G (2010). Role of media reports in completed and prevented suicide: Werther v. Papageno effects. Br J Psychiatry.

[13] SAVE (2020). Best practices and recommendations for reporting on suicide. Reporting on Suicide.

[14] Batterham PJ, Calear AL, Christensen H (2013). The Stigma of Suicide Scale. Psychometric properties and correlates of the stigma of suicide. Crisis.

[15] Whorf BL, John BC (1956). Language, Thought, Reality: Selected Writings of Benjamin Lee Whorf.

[16] Sumner SA, Burke M, Kooti F (2020). Adherence to suicide reporting guidelines by news shared on a social networking platform. Proc Natl Acad Sci U S A.

[17] Thompson A (2017). All the news.

[18] Choi D, Sumner SA, Holland KM, Draper J, Murphy S, Bowen DA, Zwald M, Wang J, Law R, Taylor J, Konjeti C, De Choudhury M (2020). Development of a machine learning model using multiple, heterogeneous data sources to estimate weekly US suicide fatalities. JAMA Netw Open.

[19] Hagar N, Wachs J, Horvát E (2021). Writer movements between news outlets reflect political polarization in media. New Media & Society.

[20] Tewari S, Zabounidis R, Kothari A, Bailey R, Alm CO (2021). Perceptions of human and machine-generated articles. Digital Threats.

[21] Sridhar S, Sanagavarapu S (2021). Content based news recommendation engine using Hybrid BiLSTM-ANN feature modelling.

[22] Pirkis J, Blood RW (2001). Suicide and the media. Part II: portrayal in fictional media. Crisis.

[23] Chancellor S, Sumner SA, David-Ferdon C, Ahmad T, De Choudhury M (2021). Suicide risk and protective factors in online support forum posts: Annotation Scheme Development and Validation Study. JMIR Ment Health.

[24] Hawton K, van Heeringen K (2009). Suicide. Lancet.

[25] Clark K, Khandelwal U, Levy O, Manning C (2019). What does BERT look at an analysis of BERT's attention.

[26] Coenen A, Reif E, Yuan A, Kim B, Pearce A, Viégas F, Wattenberg M (2019). Visualizing and measuring the geometry of BERT. Proceedings of the 33rd International Conference on Neural Information Processing Systems.

[27] Rudkowsky E, Haselmayer M, Wastian M, Jenny M, Emrich Š, Sedlmair M (2018). More than Bags of Words: Sentiment Analysis with Word Embeddings. Communication Methods and Measures.

[28] Shao Y, Taylor S, Marshall N, Morioka C, Zeng-Treitler Q (2018). Clinical text classification with word embedding features vs. bag-of-words features.

[29] Devlin J, Chang M, Lee K, Toutanova K (2019). BERT: Pre-training of deep bidirectional transformers for language understanding.

[30] Miller GA (1995). WordNet: a lexical database for English. Commun ACM.

[31] Wolf T, Debut L, Sanh V (2020). Transformers: state-of-the-art Natural Language Processing. Proceedings of the 2020 Conference on Empirical Methods in Natural Language Processing: System Demonstrations.

[32] Kwak H, An J, Jing E, Ahn Y (2021). FrameAxis: characterizing microframe bias and intensity with word embedding. PeerJ Comput Sci.

[33] Johns MM, Lowry R, Andrzejewski J, Barrios LC, Demissie Z, McManus T, Rasberry CN, Robin L, Underwood JM (2019). Transgender identity and experiences of violence victimization, substance use, suicide risk, and sexual risk behaviors among high school students - 19 states and large urban school districts, 2017. MMWR Morb Mortal Wkly Rep.

[34] Exempt IRB review. Georgia Tech Office of Research Integrity Assurance.

[35] Tzannes NS, Noonan JP (1973). The mutual information principle and applications. Inf Control.

[36] Barney LJ, Griffiths KM, Jorm AF, Christensen H (2006). Stigma about depression and its impact on help-seeking intentions. Aust N Z J Psychiatry.

[37] Niederkrotenthaler T, Reidenberg DJ, Till B, Gould MS (2014). Increasing help-seeking and referrals for individuals at risk for suicide by decreasing stigma. Am J Prev Med.

[38] Niederkrotenthaler T, Braun M, Pirkis J, Till B, Stack S, Sinyor M, Tran US, Voracek M, Cheng Q, Arendt F, Scherr S, Yip PSF, Spittal MJ (2020). Association between suicide reporting in the media and suicide: systematic review and meta-analysis. BMJ.

[39] Dempsey RC, Fedorowicz SE, Wood AM (2023). The role of perceived social norms in non-suicidal self-injury and suicidality: a systematic scoping review. PLoS One.

[40] Batterham PJ, Calear AL, Christensen H (2013). Correlates of suicide stigma and suicide literacy in the community. Suicide Life Threat Behav.

[41] Eilers JJ, Kasten E, Schnell T (2021). Comparison of stigmatization of suicidal people by medical professionals with stigmatization by the general population. Healthcare (Basel).

[42] Bohanna I, Wang X (2012). Media guidelines for the responsible reporting of suicide: a review of effectiveness. Crisis.

[43] Roth R, Abraham J, Zinzow H, Wisniewski P, Khasawneh A, Chalil Madathil K (2020). Evaluating news media reports on the 'Blue Whale Challenge' for adherence to suicide prevention safe messaging guidelines. Proc ACM Hum-Comput Interact.

[44] Nathan NA, Nathan KI (2019). Suicide, stigma, and utilizing social media platforms to gauge public perceptions. Front Psychiatry.

[45] Lee H, An S (2016). Social stigma toward suicide: effects of group categorization and attributions in Korean health news. Health Commun.

[46] Li A, Huang X, Jiao D, O'Dea B, Zhu T, Christensen H (2018). An analysis of stigma and suicide literacy in responses to suicides broadcast on social media. Asia Pac Psychiatry.

[47] Boudry V (2008). Suicide story frames contribute to stigma. Newsp Res J.

[48] Ransing R, Menon V, Kar S, Arafat SMY (2021). Artificial intelligence-based models for augmenting media reporting of suicide: challenges and opportunities. Glob Psychiatry Arch.

[49] Martínez AAB (2019). A critical literature review of the research on suicide from a gender perspective. Soc Med.

[50] Arendt F (2018). Framing suicide - investigating the news media and public's use of the problematic suicide referents Freitod and Selbstmord in German-speaking countries. Crisis.

[51] (2023). Suicide prevention resource for action. Centers for Disease Control and Prevention.

[52] Stack S (2020). Media guidelines and suicide: a critical review. Soc Sci Med.

[53] Zhang R, Wang MS, Toubiana M, Greenwood R (2021). Stigma beyond levels: advancing research on stigmatization. Acad Manag Ann.

